# Using behavioural science to enhance use of core outcome sets in trials: protocol

**DOI:** 10.12688/hrbopenres.13510.2

**Published:** 2023-11-20

**Authors:** Karen Matvienko-Sikar, Molly Byrne, Mike Clarke, Jamie Kirkham, Jan Kottner, Katie Mellor, Fiona Quirke, Ian J. Saldanha, Valerie Smith, Elaine Toomey, Paula Williamson

**Affiliations:** 1 School of Public Health, University College Cork, Cork, Ireland; 2 School of Psychology, National University of Ireland, Galway, Galway, Ireland; 3 Centre for Public Health, School of Medicine, Dentistry and Biomedical Sciences, Queen’s University Belfast, Belfast, UK; 4 Centre for Biostatistics, The University of Manchester, Manchester Academic Health Science Centre, Manchester, UK; 5 Charité-Universitätsmedizin Berlin, Institute of Clinical Nursing Science, Berlin, Germany; 6 Centre for Statistics in Medicine, University of Oxford, Oxford, UK; 7 College of Medicine, Nursing & Health Sciences, Áras Moyola, National University of Ireland, Galway, Galway, Ireland; 8 Center for Evidence Synthesis in Health; Department of Health Services, Policy, and Practice, Department of Epidemiology, Brown University School of Public Health, Providence, USA; 9 School of Nursing and Midwifery, Trinity College Dublin, Dublin, Ireland; 10 School of Allied Health, University of Limerick, Limerick, Ireland; 11 Trials Methodology Research Partnership, Department of Health Data Science, University of Liverpool, Liverpool, UK

**Keywords:** Core outcome sets, trials, trial methodology

## Abstract

**Background:**

Core outcome sets (COS) represent agreed-upon sets of outcomes, which are the minimum that should be measured and reported in all trials in specific health areas. Use of COS can reduce outcome heterogeneity, selective outcome reporting, and research waste, and can facilitate evidence syntheses. Despite benefits of using COS, current use of COS in trials is low. COS use can be understood as a behaviour, in that it is something trialists do, or not do, adequately. The aim of this study is to identify strategies, informed by behaviour change theory, to increase COS use in trials.

**Methods:**

The project will be conducted in two stages, informed by the behaviour change wheel (BCW). The BCW is a theoretically based framework that can be used to classify, identify, and develop behaviour change strategies. In Stage 1, barriers and enablers to COS use will be extracted from published studies that examined trialist’s use of COS. Barriers and facilitators will be mapped to the components of COM-B model (capability, opportunity, and motivation), which forms part of the BCW framework. Stage 2 will build on Stage 1 findings to identify and select intervention functions and behaviour change techniques to enhance COS use in trials.

**Discussion:**

The findings of this study will provide an understanding of the behavioural factors that influence COS use in trials and what strategies might be used to target these factors to increase COS use in trials.

Core outcome sets (COS) are agreed-upon sets of outcomes that should be measured and reported in all trials in specific health areas ^1^. COS are not necessarily the only outcomes that should be included in trials, but they represent the minimum set of outcomes to be measured and reported. For instance, a recently published COS for post-COVID-19 condition, included 11 outcomes such as fatigue, pain, survival, and post-exertion symptoms ^2^. COS are also applicable for use in other study designs, such as observational studies and clinical audit ^1^. COS are usually developed using input from broad stakeholders, such as researchers (including trialists), healthcare professionals, patient/public representatives, and research funders ^3^. COS are used and/or recommended for use by these stakeholders ^1, 3^. COS use involves trialists including the COS in the trial design, measuring the COS outcomes during the trial, and reporting the COS outcomes in the final trial report. Use of COS facilitates evidence syntheses ^1, 4, 5^ and can reduce outcome heterogeneity ^6^, selective outcome reporting ^7^, and research waste ^8^. Despite the benefits of using COS in trials, low COS use has been demonstrated across multiple areas of health research ^9– 12^. Low use of COS is problematic because it means that methodological improvements associated with COS are not finding their way into trial conduct quickly enough.

Previous research has examined potential reasons for low COS use in trials, including identification of barriers and facilitators to COS use among researchers with trials registered on the International Standard Randomised Controlled Trial Number (ISRCTN) Registry ^12^, researchers who submitted funding applications to the National Institute for Health Research Health Technology Assessment (NIHR HTA) programme ^13^, researchers named as chief investigators of NIHR HTA funded trials ^14^, and researchers who published a trial report in a major medical journal (e.g., *The Lancet, BMJ*) ^9^. Identified barriers to COS use include poor knowledge about existence of COS, and perceived outcome measurement issues, including patient burden ^9, 14^. Facilitators for COS use include good trialist knowledge about what COS are and how to use them, perceived importance of COS in trials, and having funder or professional recommendations or requirements to use COS in funded research ^9, 14^.

Whether or not trialists use COS in their trials can be understood as a behaviour because COS use is something trialists do, or not do. As such, COS use in trials might be modified and increased through theoretically informed behaviour change strategies. The Behaviour Change Wheel (BCW) provides a useful theoretically based framework ^15^ to identify and develop behaviour change strategies for COS use. The BCW framework includes the COM-B model, in which behaviour is predicated on an individual’s capability, opportunity, and motivation to engage in that behaviour ^15, 16^. Following the COM-B model, for a behaviour to occur, an individual must have the physical and psychological capability, social and physical opportunity, and reflective and automatic motivation to engage in the behaviour ^15, 16^. In addition to the COM-B model, the BCW also includes systematic guidance on identifying targeted intervention components and content in the form of intervention functions and behaviour change techniques (BCTs) ^15– 17^. Using the BCW is particularly useful as it facilitates mapping of barriers and facilitators to behavioural intervention functions and BCTs. As such, it can provide a systematic approach to selecting and implementing a wide range of intervention functions based on what is known about the behaviour. Adopting a behavioural science approach to understanding COS use behaviours and developing strategies to increase COS use using the BCW has the potential to maximise benefits of COS and improve the quality of trials and evidence-based practice.

The overall project aim is to identify strategies, informed by the BCW, to increase trialist use of COS in trials. The findings of this project will provide an understanding of the behavioural factors that influence COS use in trials, what strategies might be used to target these factors to increase COS use.

## Methods

We will achieve the overall project aim by examining behavioural factors identified in the previous research ^9, 12– 14^ that influence whether or not trialists use COS. The focus is on the individual trialist behaviour, rather than broader system behaviours and factors, which are being examined in a separate project ^18^. We will then map these factors to behaviour change strategies. This project will be conducted in two sequential stages to identify and prioritise behaviour change strategies that could enhance COS use in trials.

### Stage 1. Identification of behavioural barriers and facilitators to COS use

Stage 1 is informed by the first phase of the BCW, which involves understanding the behaviour to be examined (i.e., use of COS in trials). This includes defining the components of the behaviour in terms of *who, what, where, when*, and *how often* the behaviour is done. Existing data from recently published examinations of COS use ^9, 12– 14^ and research team expertise will be used to understand and define use of COS in trials for these components.

Following on from behavioural specification of COS use in trials, barriers to and enablers of this behaviour will be extracted from four recent studies (all published since 2019) that examined trialist use of COS. These papers were identified in a recent review of COS uptake as addressing why trialists use, or do not use, COS ^19^. They represent up to date research that had been conducted on use and uptake of broad cohorts of COS, rather than use of COS in specific health areas. This information across a broad cohort of health areas is needed to develop intervention strategies that are potentially useful across health areas. It is important to note that the papers report on barriers and facilitators experienced predominantly by UK and European based researchers; for instance, one paper focuses solely on UK-based researchers ^13^. This presents a limitation in terms of representativeness. However, global research on this topic was not available for inclusion. The four papers are listed below

1. A review and survey of COS use in a cohort of trials published in major medical journals ^9^.
2. A review and survey of COS use in funding applications submitted to the NIHR HTA programme ^13^.
3. A survey of trialists named as the contact person for trials registered on the ISRCTN Registry ^12^.
4. A qualitative study of trialist barriers and facilitators to COS use ^14^.

Barriers and facilitators will be extracted verbatim from the four papers and will be coded to the components of COM-B framework ^15, 16^ to identify behavioural components influencing trialist use of COS. These components include capability (physical and psychological), opportunity (physical and social) and motivation (automatic and reflective). The previously conducted qualitative study of trialist barriers and facilitators to COS use ^14^, utilised the COM-B framework to guide analysis, and so will inform the approach taken in the current study. A realist approach to coding will be taken, whereby we are seeking to identify and understand underlying mechanisms of sources of behaviour, in the form of barriers and facilitators. One investigator will conduct initial coding using a standardised coding form (Extended Data). All coding will be verified by a second investigator, with any discrepancies discussed to reach consensus, involving a third investigator as needed; all investigators involved in coding will have prior experience in coding using the COM-B framework. The findings from this coding will be synthesised narratively and using matrices, guided by COM-B as a deductive framework to identify behavioural components influencing COS use in trials.

### Stage 2. Identification of behavioural intervention strategies to enhance COS use in trials

The BCW framework ^15, 16^ will be applied to identify and select intervention functions to enhance COS use by trialists in trials. Intervention functions are ‘broad categories of means by which an intervention can change behaviour’ (e.g., incentivisation, training) ^15^. Intervention functions map on to COM-B components ^15^ and can be used in isolation or together to develop behavioural strategies. Stage 2 will use the findings of Stage 1 to identify potential intervention functions by mapping identified behavioural components to corresponding intervention functions. For example, the behavioural component ‘psychological capability’ maps on to the intervention functions ‘training’, ‘education’, and ‘enablement’ ^15^. Where multiple intervention functions are identified, we will ensure that the selected intervention functions are affordable, practical, effective/cost-effective, acceptable, safe, and equitable (the APEASE criteria) ^15^. Two investigators will independently apply the APEASE criteria to each identified intervention function, with APEASE criteria rated as ‘yes’, ‘no’, or ‘unsure’; any disagreements will be resolved by discussion, involving a third investigator as needed. Rationale for each decision made using the APEASE criteria will be also documented on the standardised APEASE template (Extended Data). Final decisions on inclusion and exclusion of intervention functions based on APEASE criteria will be made based on team review and discussion to reach consensus.

We will also identify potential intervention content in terms of BCTs. BCTs are irreducible, observable, and replicable active ingredients of an intervention designed to change behaviour that can be mapped from identified intervention functions ^15^. BCTs will be identified using the BCT Taxonomy Version 1 (BCTTv1) ^17^ and with reference to the more recently published ontology of BCTs ^20^. Each BCT will be operationalised by translating it from the BCTTv1 definition to a concrete application; for example, the BCT ‘demonstration of the behaviour’ may be operationalised as delivering a workshop for trialists demonstrating how COS can be included in trials. As with intervention functions, two investigators will independently screen BCTs using the APEASE criteria to evaluate identified BCTs based on the APEASE criteria ^15^. Rationale for each decision made using the APEASE criteria will be documented on a standardised template (Extended data) and any disagreements will be resolved by discussion, involving a third investigator as needed. As for intervention functions, decisions on inclusion or exclusion of BCTs will be based on team review and discussion to reach consensus.

### Ethical considerations

All research activities will be conducted following the University College Cork (UCC) Code of Research Conduct ethical approval and in accordance with General Data Protection Regulations (GDPR). Stages one and two do not involve any potential ethical issues because they relate to reviewing and synthesising evidence from the existing literature.

### Dissemination

This study is registered on the Open Science Framework (DOI: 10.17605/OSF.IO/GWYZS); accompanying data and materials will also be made openly available upon study completion on the Open Science Framework. The study findings will be submitted for publication in a peer-reviewed journal and disseminated through presentations to the working groups and the MRC-NIHR TMRP and the HRB-TMRN and presentations at general and domain specific conferences and events. Dissemination through platforms such as the Core Outcome Measures in Effectiveness Trials (COMET) Initiative, Harmonising Outcome Measures for Eczema (HOME) and Outcome Measures in Rheumatology (OMERACT) will be explored. Dissemination via social media will also be conducted.

## Discussion

Use of COS in trials can benefit evidence syntheses ^1, 4, 5^, in addition to reducing outcome heterogeneity ^6^, selective outcome reporting ^7^, and research waste ^8^. This project will provide information on strategies, informed by behavioural science, to enhance COS use in trials. The results will therefore provide the foundation for future methodology research and development and implementation of strategies to maximise COS use in trials.

## Study status

Stage one of the study commenced in March 2022.

## Data Availability

Open Science Framework: Enhancing COS Use in Trials, https://doi.org/10.17605/OSF.IO/BN3YQ (Matvienko-Sikar *et al.*, 2022) This project contains the following extended data: Supplementary File 1: Coding barriers and facilitators to COM-B components Supplementary File 2. Selection of Intervention Functions Supplementary File 3. Selection of Behaviour Change Techniques (BCTs) Data are available under the terms of the Creative Commons Attribution 4.0 International license (CC-BY 4.0).
