# Supplementary material for: Using behavioural science to enhance use of core outcome sets in trials: protocol
Source: HRB Open Res. 2023 Nov 20;5:23. Originally published 2022 Mar 24. [Version 2] doi: 10.12688/hrbopenres.13510.2 (PMC10682599; doi:10.12688/hrbopenres.13510.2)
Supplement: Peer review history

# Reviewer report: Reviewer response for version 2

**DOI:** 10.21956/hrbopenres.15152.r37096

**Published:** 2023-11-27

**Authors:** Oscar Castro

**Affiliations:** 1 Future Health Technologies,Campus for Research Excellence and Technological Enterprise, Singapore-ETH Centre, Singapore, Singapore

All my points have been addressed satisfactorily and have no further comments. Wishing the authors a successful project completion.

Is the study design appropriate for the research question?

Yes

Is the rationale for, and objectives of, the study clearly described?

Yes

Are sufficient details of the methods provided to allow replication by others?

Partly

Are the datasets clearly presented in a useable and accessible format?

Not applicable

Reviewer Expertise:

Health behaviour change; evidence synthesis; development and evaluation of theory-based interventions

I confirm that I have read this submission and believe that I have an appropriate level of expertise to confirm that it is of an acceptable scientific standard.

# Reviewer report: Reviewer response for version 1

**DOI:** 10.21956/hrbopenres.14741.r35734

**Published:** 2023-08-31

**Authors:** Oscar Castro

**Affiliations:** 1 Future Health Technologies,Campus for Research Excellence and Technological Enterprise, Singapore-ETH Centre, Singapore, Singapore

Thank you for the opportunity to participate in this review. The protocol paper describes a programme of research with three stages aimed at identifying theory-indicated behaviour change strategies to promote the use of core outcome sets (COS) when reporting medical research. The research described is novel and important. Authors have provided a comprehensive overview of the different research activities planned and their methodology. However, I lack specific details on the research protocol and I have also some suggestions to expand the discussion section. I hope my comments below will help authors to improve this submission.

 Introduction

- While the rationale is sound and well-presented, more information could be added to help readers understand what authors mean by ‘core outcome sets’. Introducing one or two examples would be helpful in this regard. Moreover, it would be good to clarify whether COS only apply to interventional research or also include all medical research (e.g., observational).
- Authors state that “ *The Behaviour Change Wheel (BCW) provides a useful theoretically based framework to identify and develop behaviour change strategies for COS use*”, but it is not clearly specified why is that the case. The selection of a given theoretical framework is critical as it will determine subsequent work according to the protocol, so it should be better justified. For example, a unique advantage of the BCW is that it helps intervention designers to not just better understand behaviour but also how to act upon it (i.e., provides a systematic approach on how to select and implement a wide range of intervention functions based on what is known about the behaviour).

Methods –Stage 1

- I lack further details on the four studies that will be used as part of this stage. Why have these been selected? Do they represent a sizeable portion of *all* literature on COS influences? Have these studies been conducted by the research team, or other researchers? What is the rationale to select studies published since 2019? These details are relevant as this constitutes the source data from which the entire project will be based on.
- I understand the COM-B will be used deductively to code the source data. Are authors using any specific qualitative analysis deductive approach (e.g., directed content analysis)? What are the authors’ epistemology stance when it comes to these qualitative analyses? Do authors plan to use the TDF to expand on the COM-B components and provide a more nuanced framework for coding (and later mapping of intervention functions / BCTs)?

Methods –Stage 2

- It is not clear to me why a prioritisation effort is taking place here to select between different intervention functions when you have a specific phase (stage 3) to precisely do that. Why not simply present all intervention functions to the experts as part of the consensus meeting? Please clarify.
- Regarding the rating for the APASE criteria, I understand you will use different categories (‘yes’, ‘no’, or ‘unsure’), but it is not clear how this will lead to an overall inclusion/exclusion decision (e.g., will you use the number of ‘yes’ per intervention function / BCT as a criterion?)
- When it comes to BCT definitions and labels, apart from the original taxonomy perhaps the research team wants to also cross-check the recently published ontology of behaviour change interventions (see below), as I understand some BCTs have been updated in this new resource.
  Marques, M. M., Wright, A. J., Corker, E., Johnston, M., West, R., Hastings, J., ... & Michie, S. (2023). The Behaviour Change Technique Ontology: Transforming the Behaviour Change Technique Taxonomy v1. Wellcome Open Research, 8.

Methods –Stage 3

- Although not explicitly mentioned, I understand the consensus meeting will be conducted in English, with most (all?) participants from English-speaking countries. Will this hamper the broad applicability of findings?
- Could you also please specify the expected duration of the meeting? It seems there are many aspects to be covered.
- Will the APEASE criteria will be somehow introduced here? For consistency with stage 2.

Discussion

- While I agree that the use of COS will potentially improve evidence synthesis, I also see some limitations which could be interesting to elaborate further to provide a broader picture. To my understanding, COS basically refers to a checklist of outcomes that need to be reported for a given health condition. That is, COS do not provide a controlled vocabulary for such outcomes to ensure the same labels are used consistently to describe the same outcomes. This is unlike other evidence synthesis approaches (e.g., ontologies such as the BCIO) that use pre-defined entities and have the added benefit of being computer-friendly, which could be very useful to support evidence synthesis automation and leverage on AI / machine learning. In addition, COS are focused on outcomes so do not seem to address other important aspects of clinical trials that would also benefit from standardisation and a more comprehensive reporting (e.g., population targeted, methods, etc). I understand this research is not about COS but rather the use of COS (i.e., it’s a behaviour change question rather than an evidence synthesis question), but I still think authors could use the introduction and/or the discussion to provide a broader overview of COS and highlight similarities (and differences) with other evidence synthesis improvement approaches (e.g., ontologies), as well as comment on how COS relates, or could be interoperable with, common reporting guidelines (e.g., CONSORT statement).
  Michie, S., West, R., Finnerty, A. N., Norris, E., Wright, A. J., Marques, M. M., ... & Hastings, J. (2020). Representation of behaviour change interventions and their evaluation: Development of the Upper Level of the Behaviour Change Intervention Ontology. Wellcome open research, 5.

Is the study design appropriate for the research question?

Yes

Is the rationale for, and objectives of, the study clearly described?

Yes

Are sufficient details of the methods provided to allow replication by others?

Partly

Are the datasets clearly presented in a useable and accessible format?

Not applicable

Reviewer Expertise:

Health behaviour change; evidence synthesis; development and evaluation of theory-based interventions

I confirm that I have read this submission and believe that I have an appropriate level of expertise to confirm that it is of an acceptable scientific standard, however I have significant reservations, as outlined above.

## References

[1] : The Behaviour Change Technique Ontology: Transforming the Behaviour Change Technique Taxonomy v1. *Wellcome Open Res* .2023;8: 10.12688/wellcomeopenres.19363.1 308 10.12688/wellcomeopenres.19363.1 37593567PMC10427801

[2] : Representation of behaviour change interventions and their evaluation: Development of the Upper Level of the Behaviour Change Intervention Ontology. *Wellcome Open Research* .2020;5: 10.12688/wellcomeopenres.15902.1 10.12688/wellcomeopenres.15902.1 33614976PMC7868854

# Response

**Published:** 2023-11-08

**Authors:** Karen Matvienko-Sikar

**Affiliations:** University College Cork, Ireland

**Reviewer comment 1.** Thank you for the opportunity to participate in this review. The protocol paper describes a programme of research with three stages aimed at identifying theory-indicated behaviour change strategies to promote the use of core outcome sets (COS) when reporting medical research. The research described is novel and important. Authors have provided a comprehensive overview of the different research activities planned and their methodology. However, I lack specific details on the research protocol and I have also some suggestions to expand the discussion section. I hope my comments below will help authors to improve this submission.

**Author response 1. **Thank you for your feedback on the protocol manuscript and for your useful suggestions for improvement, which have now been incorporated.

**Reviewer comment 2. **Introduction

 While the rationale is sound and well-presented, more information could be added to help readers understand what authors mean by ‘core outcome sets’. Introducing one or two examples would be helpful in this regard. Moreover, it would be good to clarify whether COS only apply to interventional research or also include all medical research (e.g., observational).

**Author Response 2**. We have now added the following to the introduction to provide more information and clarification on COS:

 “COS are not necessarily the only outcomes that should be included in trials, but they represent the minimum set of outcomes to be measured and reported. For instance, a recently published COS for post-COVID-19 condition, included 11 outcomes such as fatigue, pain, survival, and post-exertion symptoms. COS are also applicable for use in other study designs, such as observational studies and clinical audit ^1^.”

**Reviewer comment 3. **Authors state that “The Behaviour Change Wheel (BCW) provides a useful theoretically based framework to identify and develop behaviour change strategies for COS use”, but it is not clearly specified why is that the case. The selection of a given theoretical framework is critical as it will determine subsequent work according to the protocol, so it should be better justified. For example, a unique advantage of the BCW is that it helps intervention designers to not just better understand behaviour but also how to act upon it (i.e., provides a systematic approach on how to select and implement a wide range of intervention functions based on what is known about the behaviour).

**Author Response 3. **We agree that clearly explaining the choice of theoretical framework is important and have added the following to the introduction to better clarify this choice:

 “Using the BCW is particularly useful as it facilitates mapping of barriers and facilitators to behavioural intervention functions and BCTs. As such, it can provide a systematic approach to selecting and implementing a wide range of intervention functions based on what is known about the behaviour.”

**Reviewer comment 4. **Methods –Stage 1

 I lack further details on the four studies that will be used as part of this stage. Why have these been selected? Do they represent a sizeable portion of all literature on COS influences? Have these studies been conducted by the research team, or other researchers? What is the rationale to select studies published since 2019? These details are relevant as this constitutes the source data from which the entire project will be based on.

**Author 4. **The four studies included represent the research that had been conducted to the date of the protocol on use and uptake of broad cohorts of COS, rather than use of COS in specific health areas. This information across a broad cohort of health areas is needed to develop intervention strategies that are potentially useful across health areas. Some, but not all, members of the research team were involved in these papers, though the first authors of three papers (Dr Hughes, Ms Bellucci) are not involved in this project. This was not a deciding factor in inclusion of these papers, rather a reflection of the work of members of the research team in this area. Similarly, studies were not required to be published since 2019 but the studies identified were, thus providing up to date information. We have included the following for further clarification:

 “These papers were identified in a recent review of COS uptake as addressing why trialists use, or do not use, COS. They represent up to date research that had been conducted on use and uptake of broad cohorts of COS, rather than use of COS in specific health areas. This information across a broad cohort of health areas is needed to develop intervention strategies that are potentially useful across health areas.”

**Reviewer comment 5. **I understand the COM-B will be used deductively to code the source data. Are authors using any specific qualitative analysis deductive approach (e.g., directed content analysis)? What are the authors’ epistemology stance when it comes to these qualitative analyses? Do authors plan to use the TDF to expand on the COM-B components and provide a more nuanced framework for coding (and later mapping of intervention functions / BCTs)?

**Response 5. **We are not conducting specific qualitative deductive analyses beyond coding the included qualitative data to the COM-B framework. The epistemological stance taken in analysis of the qualitative data is a realist approach whereby we are seeking to identify and understand underlying mechanisms of sources of behaviour, in the form of barriers and facilitators. The COM-B was chosen as the single framework for use in this study to enable development of broad strategies, tied to specific behaviour change techniques that could be used. Future studies may build upon this work, utilising other frameworks such as the TDF, but this is not part of the current study.

 The following has been added to Stage 1 in the Methods to clarify the epistemological stance:

 “A realist approach to coding will be taken, whereby we are seeking to identify and understand underlying mechanisms of sources of behaviour, in the form of barriers and facilitators.”

**Reviewer comment 6.**

 Methods –Stage 2

 It is not clear to me why a prioritisation effort is taking place here to select between different intervention functions when you have a specific phase (stage 3) to precisely do that. Why not simply present all intervention functions to the experts as part of the consensus meeting? Please clarify.

**Response 6. **The consensus meeting (stage 3) has now been removed from the project. This has resulted from the decision to not conduct prioritisation of behavioural intervention components due to the heterogeneity in the behavioural intervention components identified. The research team felt that this heterogeneity would have limited meaningful ability to prioritise these techniques for trialists who are unfamiliar with them. Further, by providing a range of potential strategies, researchers can choose those which are best suited for their areas and COS.

**Reviewer comment 7. **Regarding the rating for the APASE criteria, I understand you will use different categories (‘yes’, ‘no’, or ‘unsure’), but it is not clear how this will lead to an overall inclusion/exclusion decision (e.g., will you use the number of ‘yes’ per intervention function / BCT as a criterion?)

**Response 7.**

 Decisions around inclusion or exclusion of intervention functions/BCTs are based on discussion as informed by the yes/no/unsure ratings. A numeric approach is not used to reach decisions, as individual criterion can result in intervention approaches being unfeasible. For instance, if an intervention is likely effective but is not affordable, it may be untenable. The following has been added to clarify this in the methods:

 “Final decisions on inclusion and exclusion of intervention functions based on APEASE criteria will be made based on team review and discussion to reach consensus.“

 “As for intervention functions, decisions on inclusion or exclusion of BCTs will be based on team review and discussion to reach consensus.”

**Reviewer comment 8. **When it comes to BCT definitions and labels, apart from the original taxonomy perhaps the research team wants to also cross-check the recently published ontology of behaviour change interventions (see below), as I understand some BCTs have been updated in this new resource.

 Marques, M. M., Wright, A. J., Corker, E., Johnston, M., West, R., Hastings, J., ... & Michie, S. (2023). The Behaviour Change Technique Ontology: Transforming the Behaviour Change Technique Taxonomy v1. Wellcome Open Research, 8.

**Response 8. **Thank you, the team are aware of the recently published ontology by Dr Marta Marques and colleagues, and this is consulted in the project. Reference to the ontology is now included in the methods section in relation to identifying BCTs.

**Reviewer comment 9. **Methods –Stage 3

 Although not explicitly mentioned, I understand the consensus meeting will be conducted in English, with most (all?) participants from English-speaking countries. Will this hamper the broad applicability of findings?

 Could you also please specify the expected duration of the meeting? It seems there are many aspects to be covered.

 Will the APEASE criteria will be somehow introduced here? For consistency with stage 2.

**Response 9. **As noted above, the consensus meeting has now been removed from the project.

**Reviewer comment 10. **Discussion

 While I agree that the use of COS will potentially improve evidence synthesis, I also see some limitations which could be interesting to elaborate further to provide a broader picture. To my understanding, COS basically refers to a checklist of outcomes that need to be reported for a given health condition. That is, COS do not provide a controlled vocabulary for such outcomes to ensure the same labels are used consistently to describe the same outcomes. This is unlike other evidence synthesis approaches (e.g., ontologies such as the BCIO) that use pre-defined entities and have the added benefit of being computer-friendly, which could be very useful to support evidence synthesis automation and leverage on AI / machine learning. In addition, COS are focused on outcomes so do not seem to address other important aspects of clinical trials that would also benefit from standardisation and a more comprehensive reporting (e.g., population targeted, methods, etc). I understand this research is not about COS but rather the use of COS (i.e., it’s a behaviour change question rather than an evidence synthesis question), but I still think authors could use the introduction and/or the discussion to provide a broader overview of COS and highlight similarities (and differences) with other evidence synthesis improvement approaches (e.g., ontologies), as well as comment on how COS relates, or could be interoperable with, common reporting guidelines (e.g., CONSORT statement).

 Michie, S., West, R., Finnerty, A. N., Norris, E., Wright, A. J., Marques, M. M., ... & Hastings, J. (2020). Representation of behaviour change interventions and their evaluation: Development of the Upper Level of the Behaviour Change Intervention Ontology. Wellcome open research, 5.

**Author response 10. **Thank you very much for this important comment. We fully agree that this is an important topic but feel it is beyond the scope of the work described in this protocol.

# Reviewer report: Reviewer response for version 1

**DOI:** 10.21956/hrbopenres.14741.r31753

**Published:** 2022-04-29

**Authors:** Nikita Nekliudov, Nina Seylanova

**Affiliations:** 1 Nuffield Department of Population Health, University of Oxford, Oxford, UK; 2 Department of Paediatrics and Paediatric Infectious Diseases, Institute of Child's Health, Sechenov First Moscow State Medical University (Sechenov University), Moscow, Russian Federation; 3 Sechenov Biomedical Science and Technology Park, Sechenov First Moscow State Medical University (Sechenov University), Moscow, Russian Federation

Thank you for the opportunity to review the manuscript entitled “Using behavioural science to enhance the use of core outcome sets in trials: protocol”. The protocol provides a comprehensive overview of the study methodology aiming to synthesise the strategies to increase the uptake of Core Outcome Sets (COS). The study addresses a well-recognised knowledge gap that impedes the dissemination and use of COS despite their potential to harmonise research activities, increasing the comparability across studies and shift the focus of research to patients. This protocol requires minor changes; all the possible limitations are rooted in the methodology of the studies collecting the data to be used in this study.

**Major comments**

1. A comprehensive approach and high quality of previously gathered data that is proposed to inform Stage 1 (Identification of behavioural barriers and facilitators to COS use) is a strength of this protocol; however, the process might benefit from considering a more global approach. While Stage 1 process is based on data from four studies (ref. 8,11-13) that are collected from majorly the UK and European researchers, endorsing more international participation at Stage 3 seems feasible. Given that different settings, funding policies, and various levels of access to medical/research facilities can influence COS use in different countries. It would be very informative if the authors could add some details on how this diversity will be achieved, or, if it is not possible, acknowledge that as a limitation.
2. The fourth component of the synthesis is a qualitative study of trialist barriers and facilitators, *“In-depth qualitative interviews identified barriers and facilitators that influence chief investigators’ use of core outcome sets in randomised controlled trials”* by Hughes *et al. *that was conducted on a UK sample. Despite the study methodology of the study, its limited generalisability due to possible missed barriers and facilitators relevant to trialists outside of the UK might need to be acknowledged in the relevant section of the protocol.
3. Given the primary objective of the project to release the strategies for increasing COS uptake, dissemination is an integral step to implement these strategies. Please consider expanding the dissemination strategies by including additional platforms (e.g. the COMET initiative, possibly HOME and OMERACT).

**Minor comments**

1. Authors are mentioning 10-15 participants of the consensus meeting ( *'with recruitment focused on diversity of expertise and representativeness across stakeholder groups'*) and it could be useful for readers to know the distribution across the stakeholder groups at the consensus meeting. It seems that this data can be added at this stage as the participants are going to be identified by the study group (' *Healthcare professionals and patient/ public representatives will be identified from existing networks, trials and/or outcomes research by the study team and invited to participate via direct email contact').*
2. It is stated that during the consensus meeting ( *'...a final round of ranking will follow with the aim of reaching consensus on a prioritised list of strategies to target in future research')* the consensus will be reached using the NGT approach and the process will follow COMET guidance ( *'The prioritisation meeting will be conducted using the Nominal Group Technique (NGT) approach and following recent guidance from the Core Outcome Measures for Effectiveness Trials (COMET) Initiative'*). It could be useful to state consensus criteria/prioritisation process details.

Is the study design appropriate for the research question?

Yes

Is the rationale for, and objectives of, the study clearly described?

Yes

Are sufficient details of the methods provided to allow replication by others?

Yes

Are the datasets clearly presented in a useable and accessible format?

Not applicable

Reviewer Expertise:

Epidemiology, Global Health Science, Clinical Informatics

We confirm that we have read this submission and believe that we have an appropriate level of expertise to confirm that it is of an acceptable scientific standard.

# Response

**Published:** 2023-11-08

**Authors:** Karen Matvienko-Sikar

**Affiliations:** University College Cork, Ireland

**Reviewer comment 1. **Thank you for the opportunity to review the manuscript entitled “Using behavioural science to enhance the use of core outcome sets in trials: protocol”. The protocol provides a comprehensive overview of the study methodology aiming to synthesise the strategies to increase the uptake of Core Outcome Sets (COS). The study addresses a well-recognised knowledge gap that impedes the dissemination and use of COS despite their potential to harmonise research activities, increasing the comparability across studies and shift the focus of research to patients. This protocol requires minor changes; all the possible limitations are rooted in the methodology of the studies collecting the data to be used in this study.

**Author response 1. **Thank you for your review of our protocol manuscript. We have incorporated your feedback and are grateful for your suggestions.

**Major comments**

**Reviewer comment 2. **A comprehensive approach and high quality of previously gathered data that is proposed to inform Stage 1 (Identification of behavioural barriers and facilitators to COS use) is a strength of this protocol; however, the process might benefit from considering a more global approach. While Stage 1 process is based on data from four studies (ref. 8,11-13) that are collected from majorly the UK and European researchers, endorsing more international participation at Stage 3 seems feasible. Given that different settings, funding policies, and various levels of access to medical/research facilities can influence COS use in different countries. It would be very informative if the authors could add some details on how this diversity will be achieved, or, if it is not possible, acknowledge that as a limitation.

**Author response 2. **This is a valid and important point regarding the representativeness of the data underpinning this study. We acknowledge that it is limited in terms of a more global approach and have included this as a limitation in the methods as follows: “It is important to note that the papers report on barriers and facilitators experienced predominantly by UK and European based researchers; for instance one paper focuses solely on UK-based researchers ^12^ . This presents a limitation in terms of representativeness. However, global research on this topic was not available for inclusion.”

**Reviewer comment 3. **The fourth component of the synthesis is a qualitative study of trialist barriers and facilitators, “In-depth qualitative interviews identified barriers and facilitators that influence chief investigators’ use of core outcome sets in randomised controlled trials” by Hughes et al. that was conducted on a UK sample. Despite the study methodology of the study, its limited generalisability due to possible missed barriers and facilitators relevant to trialists outside of the UK might need to be acknowledged in the relevant section of the protocol.

**Author response 3. **This point has been made clearer in the methods section in the note regarding representativeness of the data:  “…for instance one paper focuses solely on UK-based researchers ^12 ^”

**Reviewer comment 4. **Given the primary objective of the project to release the strategies for increasing COS uptake, dissemination is an integral step to implement these strategies. Please consider expanding the dissemination strategies by including additional platforms (e.g. the COMET initiative, possibly HOME and OMERACT).

**Author response 4. **We agree that broadening the dissemination is important to support use of the strategies. We have included the following text to the dissemination section: “…and presentations at general and domain specific conferences and events. Dissemination through platforms such as the Core Outcome Measures in Effectiveness Trials (COMET) Initiative, Harmonising Outcome Measures for Eczema (HOME) and Outcome Measures in Rheumatology (OMERACT) will be explored. Dissemination via social media will also be conducted.”

**Minor comments**

**Reviewer comment 5. **Authors are mentioning 10-15 participants of the consensus meeting ('with recruitment focused on diversity of expertise and representativeness across stakeholder groups') and it could be useful for readers to know the distribution across the stakeholder groups at the consensus meeting. It seems that this data can be added at this stage as the participants are going to be identified by the study group ('Healthcare professionals and patient/ public representatives will be identified from existing networks, trials and/or outcomes research by the study team and invited to participate via direct email contact').

 It is stated that during the consensus meeting ('...a final round of ranking will follow with the aim of reaching consensus on a prioritised list of strategies to target in future research') the consensus will be reached using the NGT approach and the process will follow COMET guidance ('The prioritisation meeting will be conducted using the Nominal Group Technique (NGT) approach and following recent guidance from the Core Outcome Measures for Effectiveness Trials (COMET) Initiative'). It could be useful to state consensus criteria/prioritisation process details.

**Author response 5. **The consensus meeting has now been removed from the project. This has resulted from the decision to not conduct prioritisation of behavioural intervention components due to the heterogeneity in the behavioural intervention components identified. The research team felt that this heterogeneity would have limited meaningful ability to prioritise these techniques for trialists who are unfamiliar with them.
